# Carbon-Based Nanomaterials/Allotropes: A Glimpse of Their Synthesis, Properties and Some Applications

**DOI:** 10.3390/ma11020295

**Published:** 2018-02-13

**Authors:** Salisu Nasir, Mohd Zobir Hussein, Zulkarnain Zainal, Nor Azah Yusof

**Affiliations:** 1 Materials Synthesis and Characterization Laboratory (MSCL), Institute of Advanced Technology (ITMA), Universiti Putra Malaysia, 43400 Serdang, Selangor, Malaysia; 2 Department of Chemistry, Faculty of Science, Federal University Dutse, 7156 Dutse, Jigawa State, Nigeria; 3 Department of Chemistry, Faculty of Science, Universiti Putra Malaysia, 43400 Serdang, Selangor, Malaysia; zulkar@upm.edu.my (Z.Z.); azahy@upm.edu.my (N.A.Y.)

**Keywords:** carbon nanostructures, synthesis, properties, applications, materials science

## Abstract

Carbon in its single entity and various forms has been used in technology and human life for many centuries. Since prehistoric times, carbon-based materials such as graphite, charcoal and carbon black have been used as writing and drawing materials. In the past two and a half decades or so, conjugated carbon nanomaterials, especially carbon nanotubes, fullerenes, activated carbon and graphite have been used as energy materials due to their exclusive properties. Due to their outstanding chemical, mechanical, electrical and thermal properties, carbon nanostructures have recently found application in many diverse areas; including drug delivery, electronics, composite materials, sensors, field emission devices, energy storage and conversion, etc. Following the global energy outlook, it is forecasted that the world energy demand will double by 2050. This calls for a new and efficient means to double the energy supply in order to meet the challenges that forge ahead. Carbon nanomaterials are believed to be appropriate and promising (when used as energy materials) to cushion the threat. Consequently, the amazing properties of these materials and greatest potentials towards greener and environment friendly synthesis methods and industrial scale production of carbon nanostructured materials is undoubtedly necessary and can therefore be glimpsed as the focal point of many researchers in science and technology in the 21st century. This is based on the incredible future that lies ahead with these smart carbon-based materials. This review is determined to give a synopsis of new advances towards their synthesis, properties, and some applications as reported in the existing literatures.

## 1. Introduction

Carbon is unique and an indispensable element in our world; it is the sixth most common element in the universe and the 4th most common element in the solar system and 17th most common element in the Earth’s crust [1]. It has been estimated that the relative abundance of carbon is between 180 and 270 parts per million [2]. Remarkably, it is also the second most common element in the human body after oxygen [3], thus taking/making about 18 percent of a human’s body weight. One of the outstanding descriptions of carbon is being of a broad range of metastable phases that can be formed near ambient conditions and their spacious fields of kinetic stability.

Although, elemental carbon is sparse on the earth’s crust with barely 0.2% of the total mass of this planet [1,2,4], however, its function is incredibly essential as it can form bonds with other light elements and itself. As a result, the ability of carbon to catenate paved the way on which chemistry and biology have been expanded, and eventually making the wonders of life to occur [3]. As such, carbon science is very trendy today and in the field of nanoscience, materials science, engineering and technology, carbon nanostructures are identified to comprise of different low-dimension allotropes of carbon including graphite, activated carbon, carbon nanotubes, and the C_60_ family of buckyballs, polyaromatic molecules [5,6,7,8] and graphene [9,10,11]. In the contemporary period, nanotechnology has attracted substantial attention due to its direct application to generate new materials with exclusive properties. Many factors such as superior directionality, high surface area and flexibility make nanostructures suitable for a wide range of applications [12,13,14,15,16,17]. This is the reason why researchers from various scientific backgrounds are very much curious in these materials taking into consideration the key role they have played in many new advanced technologies. This ability opens up many new areas of chemistry for nanomaterials design, including the growth of functional nanoparticle arrays for catalytic applications [8,18,19], the selective sequestration of chemicals for drug delivery [11,18,20,21,22,23], melting of phase change materials (PCM) by thermal conductivity enhancer (TCE) in building prototype [24,25,26,27,28,29] and the creation of mesoporous monolithic structures as low-k dielectric materials [10,30,31], etc.

In this review, we will discuss not only the recent investigations in these areas, but also some other new interesting applications will be incorporated. Primarily, we will focus on the properties and how these carbon nanomaterials can be synthesized and tailored towards specific applications more particularly electrochemical energy storage.

### 1.1. Historical Chemical Background of Some Selected Carbon-Based Allotropes

**Figure 1:**
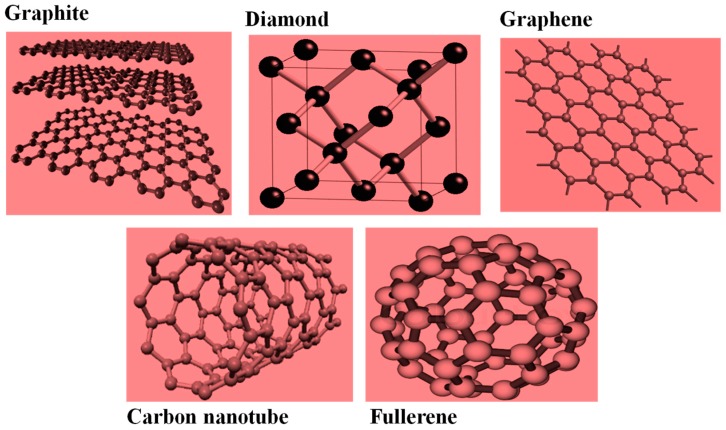
Structural illustration of some 0-, 1-, 2- and 3-dimensional carbon nanomaterials with sp^2^ and sp^3^ hybridization allotropes occurring in different crystallographic forms [44].

For a very long period of time, carbon is conventionally known to exist in two natural crystalline allotropic forms commonly known as graphite and diamond. However, the chemistry of these two substances differs more importantly in crystal structures and properties [32,33,34,35,36,37,38]. Chemically speaking, many other allotropes can be formed due to the valence of carbon atoms. This occurred when carbon atoms form covalent bonds with another carbon atom [39]. To easily understand this phenomenon, allotropes are elements which are chemically identical but vary strikingly in their physical properties. Therefore, when the atoms in substance that have only one type of atom are organized in a different way than an allotrope is established. For this reason, several other allotropes and forms of carbon were discovered (Figure 1) such as graphene [10], buckminsterfullerene [40,41], carbon nanotubes [42,43], etc., hence making carbon to have the highest number of identified allotropes when compared to any other material.

### 1.2. Graphite

The word graphite was reported to have been derived from the primordial Greek word ‘graphein’ [45]. It was named by Abraham Gottlob Werner in 1789 and consists of carbon atoms connected together in huge flat networks that are piled on top of each other. This allotrope of carbon is an excellent electrical conductor making it a very good material for electrode in an electrical arc lamp [46]. The ability to conduct electricity occurs as a result of delocalization of π-electrons of the carbon atoms in graphite, a phenomenon that is not feasible in diamond. Hence, diamond cannot conduct electricity due to the restricted movement of the electrons in its lattice arrangement [47]. It has been established that graphite is the most stable form of carbon under standard conditions. The first synthetic graphite was produced by an American scientist, Edward Acheson in 1896.

The material (graphite) is characterized with a marked lustrous black sheen feature and experimentally tested to be very flexible but non-elastic. It is also known to possess the properties of both metals and non-metals, with high thermal and electrical conductivity, chemically inert and physically greyish-black and opaque in nature [47,48,49]. The basis for all of the aforementioned remarkable properties could be attributed to its crystal structure. It is interesting to also note that the carbon atoms in graphite are structurally arranged hexagonally in a planar condensed ring system.

**Figure 2:**
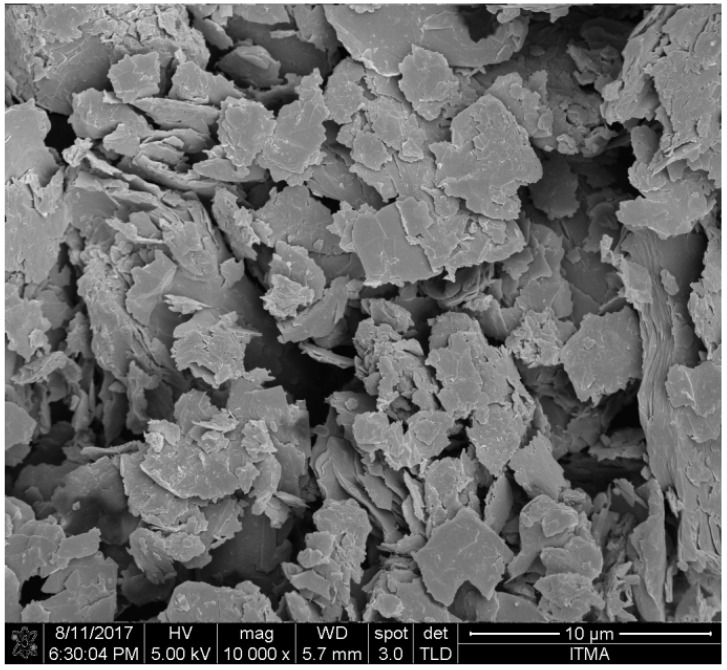
Field emission scanning electron and polarized light micrographs of the platy morphology of flake graphite.

Moreover, the layers are piled parallel to each other and a covalent bond strongly held the atoms together within the rings. On the contrary, the layers are slackly bonded together by van der Waals forces. Based on this inter-layer weakness caused by the attractive part of the van der Waals forces, the layers are therefore capable to move past each other and this is the reason for soft and slippery physical property of graphite, which in effect make it a good lubricating material in dynamos and electric motors. In summary, graphite can be found mainly with a flaky morphology as shown in Figure 2 below. Despite the difficulty of preparing graphite nanoparticles or nanosheets, however, in 2002 a new process was successfully developed that was effectively used to exfoliate natural flake graphite into nanosheets with thickness ranging from 30 to 80 nm [50].

**Figure 3:**
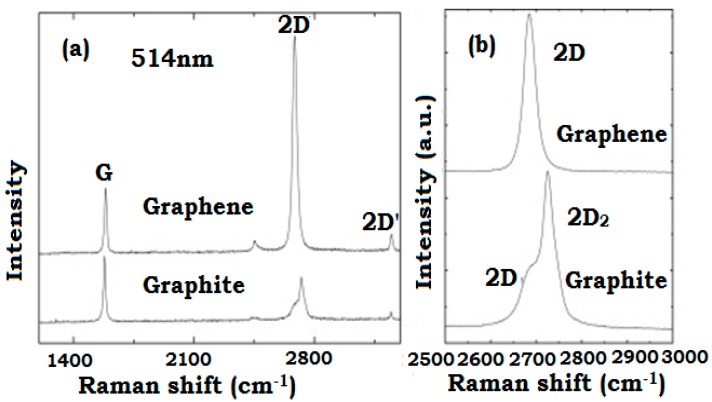
(**a**) Raman spectra of graphene as compared to that of graphite measured at 514.5 nm; (**b**) comparison of the 2D peaks in graphene and graphite. Reproduced with permission from [51].

The advancement of research related to ‘grahenic-like carbons’ was primarily connected to the utility and versatility of Raman spectroscopy. This technique is essentially useful for distinguishing various graphenic forms of carbon. For example, in Figure 3, graphite (which is a stacked structure of graphene) can be distinguished from the actual graphene plane by their Raman spectra. Despite the fact that the spectra looks somehow alike due to the fact that both materials involve graphene sheets, yet, some considerable differences could be identified, particularly on their first overtone of D band (2D), also known as G’ band [51]. It is noteworthy that both 2D and G’ bands are the conventional names for bands at around 2650 cm^−1^ on the Raman spectra. The 2D band shape and position is used to distinguish different number of layers and for this reason, it can be used to easily differentiate graphene from graphite [51,52,53,54]. Equally important is that the technique is very responsive to defects in aromatic compounds and thus able to easily assess the quality of graphene. It has been observed that the peak shift in graphite spectra results from the interactions among the stacked graphene layers which are believed to change the band’s position with elevated frequency [51,55].

As for graphene and graphite, the Raman spectroscopy has been widely used for the characterization of carbon nanotubes, fullerenes and other allotropes forms of carbon. It is worth noting that the Raman spectra of carbon nanotubes contain G, D, and 2D bands and the prominent radial breathing modes. Due to the resonant mechanism involves in the Raman spectroscopy, the said radial breathing modes frequencies and intensities is caused due to the nanotube electronic structure which is itself governed by the way the graphene plates are rolled. In this light, resonant Raman spectra for a wide excitation energy region were obtained by Kataura in the late 1990s [56] and the plot has become a means that can provide the intensity of a given mode in function of the laser used to carry out the Raman spectroscopic measurements.

Looking back at the historical background of Raman spectroscopy, an Indian scientist Sir Chandrasekhara Venkata Raman (1888–1970) and co-workers were the first scientists to observe in practice the inelastic scattering of light in 1928 [57]. Therefore, the Raman Effect owes its name to C.V. Raman and the discovery paved the way for him to win the Nobel Prize in Physics in 1930 [58]. This breakthrough was accomplished using sunlight, a narrow band photographic filter to create monochromatic light, and a “crossed filter” to block this monochromatic light. Fascinatingly, he observed that a small amount of light had changed frequency and penetrated through the crossed filter. In a nutshell, the Raman spectroscopy explores molecular and crystal lattice vibrations and therefore is sensitive to the phase, chemical environment, composition, bonding and crystalline structure of the analyte (sample) material. These characteristics make it an excellent technique for identifying materials in any physical form, including solid such as (crystalline or amorphous) [59], liquids, gases and solutions [60]. Since its discovery, Raman spectroscopy has become a modus operandi and an established means for the characterization of different types of materials particularly carbon-based materials over the past decades.

It is worth noting that the technique is generally susceptible to highly symmetric covalent bonds with slight or no natural dipole moment. The carbon–carbon bonds that make up these materials were found to perfectly go well with this criterion and as a result the Raman spectroscopy is extremely sensitive to these materials and thus capable to give vital information about their structure. It is also suitable for examining chemical changes under some physicochemical process and to measure mechanical stress or stress release. More so, the technique is also applicable in determining the electronic properties, diameter of carbon nanotubes, coupling between a carbon phase and another environment [61], etc.

Equally important, the technique is capable of detecting even diminutive structural changes and in all cases provides information on defects, thus making it a very important analytical tool for the characterization of carbon nanomaterials [61].

### 1.3. Diamond

Just like graphite, diamond has been known to exist since ancient times. However, its usage was only limited to decorations at that time, which is now perceived by the contemporary scientists as a misuse of its appropriate potential. As one of its popular excellent properties, diamond is known to have the highest thermal conductivity when compared to any other material [62,63]. This is decisively attributed to low phonon scattering and strong covalent bonding that held its atoms. The thermal conductivity of natural diamond was reported to be approximately 2200 W/(mK), which is five times more than copper [30,64,65]. Based on the high thermal conductivity of diamond, it is widely used in the semiconductor industry to prevent silicon and other semiconducting materials from overheating [33,66]. The industrial or commercial production of synthetic diamonds traced its history in the 1950s by General Electric. It is well established that the diamond has an excellent carrier mobility, saturated carrier velocities and electric field breakdown strength [67]. However, It has a poor dielectric constant and may likely show ‘negative electron affinity’ when tested. Many reports considered it to be a wide band gap semiconductor with an eV value of 5.5 [68] that can be doped to *p*-type or *n*-type. Based on its physicochemical properties, diamond is chemically and physically robust, and radiation ‘hard’. It is therefore believed that any electronics device made from diamond should not only perform at the highest levels, but should also be capable of operating in extreme environments [16,65,66]. Its optical properties are usually considered uncommon and been genetically linked with the carbon family [64,69,70], it is considered to be biocompatible inside a living body. Thus, it is not prone to unwanted cell adhesion or particulate generation. In relation to its thermal stability, diamond (as a form of carbon) can easily be oxidized in air when heated beyond 700 °C. However, in a flow of high purity argon gas or rather in the absence of oxygen, it can be heated up to 1700 °C. Shatskiy et al. [71] reported that the material can endure a temperature of 3000 °C or even higher.

In general, those combined properties paved the way for industrial applications of diamond in windows, cutting and polishing tools, heat spreaders, and the scientific applications as an optical detector material, diamond anvil cells, diamond knives, etc.

### 1.4. Graphene and Graphene-Derived Materials (‘Graphenoids’)

**Figure 4:**
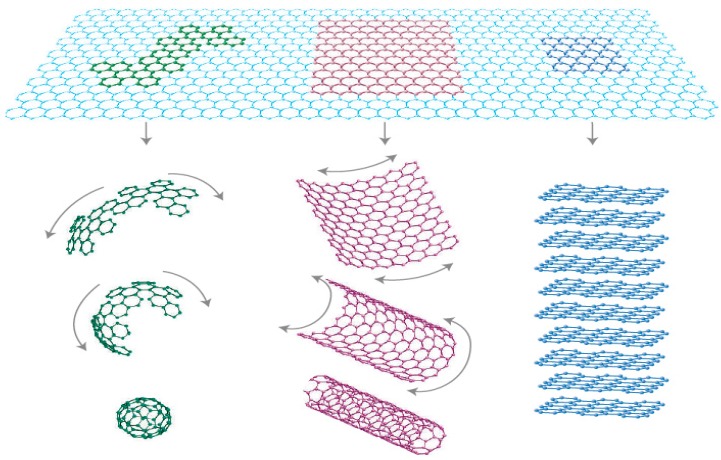
Structure of graphene configured to buckyballs (0-dimensional) by wrapping up, to nanotubes (1-dimensional) via rolling and to graphite (3-dimensional) by stacking. Reproduced with permission from [10]. Copyright nature materials.

Since its discovery at the University of Manchester by Andre Geim and Novoselov in 2004 [72], graphene has become a material of interest and absolutely alerted the scientific curiosity of many research communities around the world. Therefore, it is not an exaggeration to say graphene is one of the most researched, speculated and promising nanomaterials in the past decade. This is based on its exclusive combination of wonderful properties which are inconceivable to normal materials, and this paved the way for its exploitation in a large variety of applications [10,11,72]. Many important carbon materials contain graphene as the primary building block of their structure. For example, a stacked graphene gives graphite and a rolled-up graphene sheets give carbon nanotubes (Figure 4) [10]. It has been practically proven that the quality of produced graphene has a direct effect on its electronic and optical properties. Therefore, presence of defects, impurities, structural disorders and wrinkles in the graphene sheet can adversely affect those properties [51,73,74,75]. Consequently, in electronic applications, it is imperative to have high quality and single crystalline graphene thin films with high electrical and thermal conductivities alongside with dazzling optical transparency [11,73,75,76,77,78,79,80].

A widespread and commercial application of graphene and graphene-related materials has been hindered to some extent by the high cost towards the production of these materials. Hence, a number of researchers have used materials that are generally cheap such as agricultural waste (bio-waste) [81,82,83], insects, food, etc. as the precursors for the synthesis of carbon nanomaterials particularly graphene [7,84] (Table 1).

A report entitled “The Global Market for Graphene to 2017” by the Future Markets, Inc. (Edinburgh, UK) 2012 revealed that the production volume of graphene in 2010 was 28 tones and is predicted to grow to 573 Tones by the end of 2017 [85].

### 1.5. Activated Carbon

By considering its nature, properties and forms, activated carbon (AC) is placed or rather fit into the amorphous carbon category. It is believed to be the most popular and established of all carbons fabricated from sustainable resources [86]. Chemically speaking, AC is a carbon that has been chemically enhanced to micro porous structure. The surface functionality in AC results in materials that are excellent at adsorption of various chemical species. It is popularly known to possess a high surface area to volume ratio, which in turn make it very useful as an adsorbent material [87,88]. Therefore, it is commonly used as an adsorbent in poisoning, reducing cholesterol level, plummeting internal gas, flatulence and many other useful applications [89,90,91]. Historically, in the previous century, activated carbon was mainly used for purifying air and water supply and demand for it grew rapidly. In the 1950s, the invention of carbon fibers paved the way for a new lightweight and incredibly strong material.

Conventionally, activated carbon are mostly produced from biomass precursors such as lignocellulosic materials (palm kernel shell, oil palm trunk, olive cake, olive bagasse, oil palm empty fruit bunch, wood, coconut shells, date palm seeds, rice husk etc.) [87,88,89,90,91,92,93,94,95,96,97], different kinds of coal or other sorts of carbon [24,86], etc. (Table 2). The production procedure usually requires annealing the starting raw material under reducing atmosphere to produce a carbonaceous surface. To further boost the material’s surface, the operation is subsequently followed by activation through chemical oxidation or thermal treatment. It is worth noting that not all activated carbons are fabricated using the same method and has the same physicochemical properties. Many factors such as variations in the starting materials, different methods of activation and operational conditions, etc. can generate a carbon material with a spacious collection of surface properties. These properties are believed to be directly linked to the pore size distribution in the activated surface and the kinds of functional groups present in the pores [93,94,96,97,98,99,100,101,102,103,104].

A report from the global activated carbon market shows that between 2007 and 2012, the price of AC on the global market experienced rapid growth. Similarly, a remarkable increase was also recorded in 2013. For example, in 2013 alone, the global AC market was about 2.23 billion USD and the market was projected to keep on growing at an average compound rate until 2018 due to strict government policies on mercury removal in power plants.

### 1.6. Fullerene, Buckyball and Carbon Nanotubes Family

The discovery of C_60_ was published in nature in November 1985 by Harry Kroto and Richard Smalley of University of Sussex and Rice University, respectively. It was this report, which paves the way for fullerene-related carbon nanotube synthesis. Fullerenes or bulkyballs composed exclusively of carbon of varying size and molecules which resembles a hollow sphere or tube [40]. Fullerenes have been studied comprehensively in relation to carbon nanotubes, and together with the latter have opened a new science and technology field on nano-scale materials since before the advent of graphene in 2004. Despite the fact that fullerenes and carbon nanotubes have an indistinguishable range of diameters, however, the materials are expected to exhibit assorted kinds of size effects on their properties as well as their structures. It is commonly known that fullerenes and carbon nanotubes are zero- and one-dimensional materials, respectively; this brings various effects to instigate on their structures and properties.

As the word ‘nanotube’ entails in the name of all sorts of carbon nanotubes, the materials consist of only two coaxial cylinders. The multi-walled sort of nanotubes has an outer diameter as small as 55 Å and an inner diameters as small as 23 Å [43]. In a simple term, carbon nanotubes possess a thickness or diameter on the order of only some nanometers and the thickness is comparatively about 50,000 times smaller than the width of a human hair and can be up to many centimeters length-wise. Nanotubes belong to the fullerene structural family, which also comprises buckyball. Structurally, a nanotube is cylindrical in shape with one of its ends usually wrapped into a hemisphere of buckyball spherical structure.

Basically, carbon nanotubes are divided into two main categories; single-walled nanotubes (SWNTs) and multi-walled nanotubes (MWNTs). Experimental studies reveal that nanotubes are the stiffest and strongest fibers ever produced [105]. This has been notably correlated with better mechanical and electronic properties of this material. An excellent nanotube was reported to have a Young’s modulus of as high as 1000 GPa, which is about five times higher than steel, while their tensile strength can be up to 100 GPa, nearly 50 times higher than steel which is an excellent quality for structural application when coupled with their lower density [7]. In order to maximally harness these properties tailored for specific applications, there is a need to incorporate the tubes into nanocomposite materials. In light of the above, for instance, in drug delivery systems, carbon nanotubes have been found to be proficient to improve metabolism of drugs and increase their therapeutic effect and decrease toxicity of bioactive materials by conjugating with therapeutic molecules which as a result facilitate those molecules to unravel some of their intrinsic drawbacks [106].

## 2. Synthesis

Nowadays, a lot of methods were developed to fabricate carbon nanomaterials. However, the most interested ones in recent times are using energy saving reactions and low-cost starting materials.

**Figure 5:**
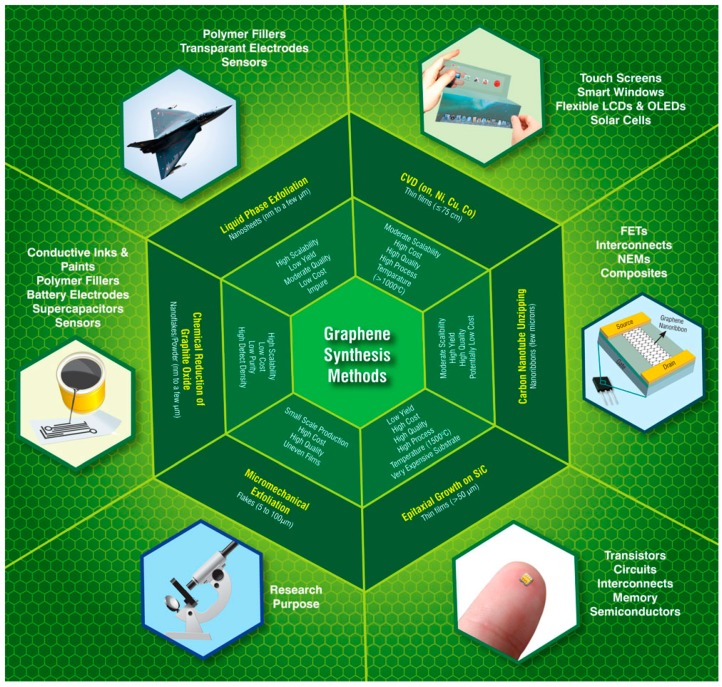
A schematic diagram illustrating the main processes frequently employed for the preparation of graphene along with their key features, and the existing and prospective applications. Reproduced with permission from [107].

Here, we begin with the most speculated and researched nanomaterials, graphene in the past decade. It should be recalled that since its isolation in 2004, several methods have been employed for the manufacture of graphene (Figure 5) which includes micromechanical cleavage [108], epitaxial growth on SiC substrates [109], chemical reduction of exfoliated graphene oxide [110], chemical vapor deposition (CVD) [111], liquid phase exfoliation of graphite [112] and unzipping of carbon nanotubes [113], etc. Each method has proven its merits and limitations, respectively, depending on its intended application. The influence of graphene as the future material for the fabrication of lightweight, miniaturized, ultra fast and high frequency electronic and optoelectronic devices has been forecasted as a brighter one. However, this can only be achieved if the quality of the 2D material is not compromised during its production. Therefore, the most suitable form of this material for this kind of applications is considered to conform with a few layers thin films of large area graphene with great domain size and consistent thickness, absolutely pure and devoid of any form of structural disarray. For this reason, the only method that can be considered suitable to fabricate graphene with most of the qualities mentioned above is by the use of chemical vapor deposition (CVD).

Another important material in the graphene family is graphene oxide and it is predominantly regarded as the most used substance to produce graphene-like material on a large scale [114]. The oxidation of graphite flakes to generate a non-stoichiometric compound (graphite oxide) was originally reported by Brodie in 1859 [115]. Since then, considerable attention was given to oxygenated graphite due to its inconceivable thermal, mechanical and electrical properties [116,117]. The existence of reactive oxygen functional groups in the GO molecule is considered to be the reason for all these outstanding properties. For this reason, the material is now used in many types of applications such as insulating materials (due to the disrupted sp^2^ bonding networks) [118,119,120], sensors [80], polymer composites [121,122], field effect transistors and energy related materials [123,124]. In recent years, it is also used in biomedical applications [22,23] due to its exceptional aqueous processability, surface enhanced Raman scattering fluorescence quenching ability and surface functionalization potential.

During GO synthesis, it has been proven difficult to control and measure the relative proportions of the elements that take part in the chemical reaction. Therefore, the stoichiometry and the initial oxygen concentration all depend on the processing condition. Consequently, factors such as elemental composition, particle size and the nature of the graphite, synthesis procedure, duration of oxidation and the oxidizing agent used are believed to be the drivers that lead to the variations in the functional group density during production of GO [118]. The graphene oxide when exposed to a reducing agent normally produces a conductive material in a form of reduced graphene oxide (rGO). Therefore, this material produced when the oxygen functional groups in the GO are removed (i.e., reduced) has a notable characteristic similar to pristine graphene. A literature survey on the preparation of graphene and its derivatives as thoroughly described in Table 1 has indicated that different synthetic pathways can be used to synthesize carbon nanomaterials (with an excellent physicochemical properties) from various waste materials at a low cost and using energy saving reactions in some cases. 

For carbon nanotubes, both SWCNT and MWCNT are fabricated by almost the same method. However, the only distinction may arise on the usage of the metal catalyst which is essentially needed for the synthesis of fullerenes. Several carbon precursors, including xylene, acetylene, toluene, methane, benzene etc. have been used as a carbon source to synthesize carbon nanotubes. However, it is alarming that these carbon feed stocks are fossil fuels-based materials; therefore, they are not renewable and sustainable.

**Figure 6:**
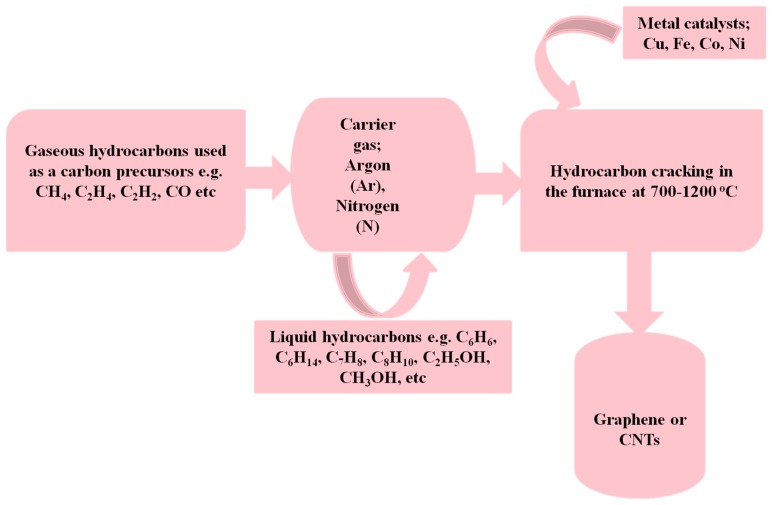
Metal compounds that are commonly used as the catalyst to synthesize single-walled carbon nanotubes (SWCNTs) and multi-walled carbon nanotubes (MWCNTs).

The metal compounds that are commonly used as the catalyst to synthesize SWCNTs and MWCNTs are shown in Figure 6 below.

We have briefly mentioned earlier that AC carbons are generally prepared in two steps. These steps are carbonization of the starting raw materials and followed by carbon activation through chemical or physical methods. In the carbonization step or process, the raw materials are thermally decomposed, removing all other species that are non-carbon and creating fixed carbon mass with a very small pore structure. The other step (activation method) is usually carried out to enhance the diameters of the small pores by increasing the area and also to generate new pores [86,102]. It is normally achieved through chemical or physical means. Chemical activation is generally accomplished through thermal disintegration of the precursor impregnated with mostly KOH, H_3_PO_4_, ZnCl_2_, HNO_3_, H_2_SO_4_, NaOH, etc. [131].

However, physical activation also popularly known as thermal activation is conducted using an oxidizing gas CO_2_ to activate the carbon material after carbonization in the temperature range between 800 to 1100 °C. The analytical pyrolysis or carbonization of the material is usually conducted in a tube furnace, muffle furnace and glass reactors sited in a modified microwave oven [132,133]. Currently, the majority of the precursors used in the production of activated carbon are mostly originated from lignocellulosic materials such as wood, coconut shells, palm oil wastes, etc. Table 2 below demonstrates the carbonization and activation conditions of various agricultural residues to prepare activated carbons.

## 3. Properties

In Table 3 and Table 4, a variety of properties of some selected carbon nanomaterials were presented. The uniqueness of carbon nanostructures (especially graphene) that attracted attention of the scientific communities is directly linked to its intrinsic strength, confirmed to exceed that of any other material [153], which is about 200 times that of steel but yet is malleable. Additionally, it is about 97% transparent when pure [154], very stable, and chemically inert materials with an abundant surface area that can be stretched by about 20% [155]. This and many other qualitative properties made graphene a ‘very good candidate’ more especially as energy storage material.

Several other derived materials such as graphene oxide (GO), reduced graphene oxide (rGO), graphene quantum dots (GQDs), etc. are considered as a derivative of graphene family. Over the years after its discovery in 1859, the GO has undergone a series of modification by a number of scientists, including Staudenmaier [156], Hummers and Offeman [116] in 1898 and 1958, respectively. When completely oxidized, the modified GO has turbostratic random ordering with 6-fold symmetry of stacking and an interlayer spacing of 0.625 nm. The high water affinity of the GO made it possible to intercalate H_2_O molecule between the layers with interlayer spacing between 6.4 nm to 11.3 Å [157,158]. As a result, the GO affinity for water make it suitable for electronic applications as it can be evenly deposited onto the substrate surface inform of thin films. Consequently, for commercial production of modified graphene-like structure with well-situated mechanical, optoelectronic, electronic and transport properties, GO is believed to be one of the most appropriate precursor based on the aforementioned properties [159]. 

## 4. Applications

**Figure 7:**
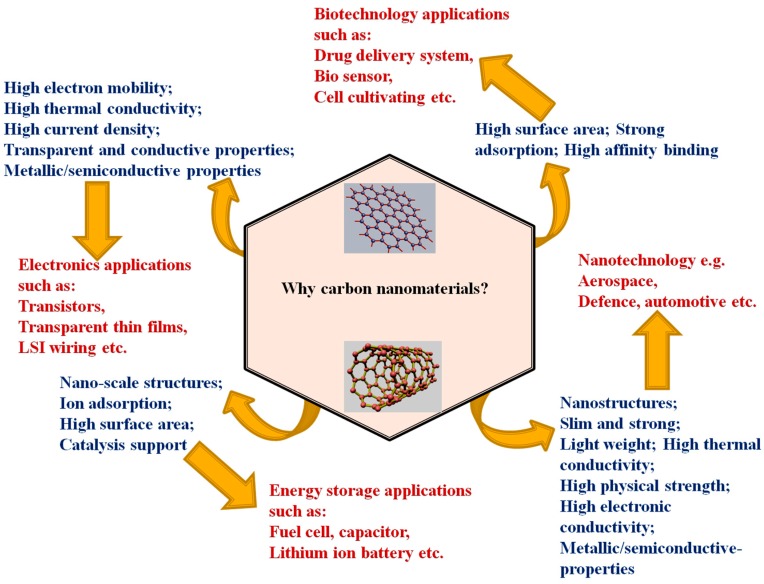
Various types of applications of carbon nanomaterials in relation to their properties. Properties were given in blue text and applications in red.

The field of material science and nanotechnology has witnessed a significant expansion in recent years across virtually all industrial sectors. This is due to a number of key advantages that traditional manufacturing cannot offer. These include mass customization, geometrical complexity, etc. Among the applications as summarized in Figure 7 are but not limited to medical [161], aerospace, defense components, household and energy related applications [29], automobile, etc. [162,163]. The application of carbon nanomaterials in the global energy scene is a topic that attracted great attention in the recent time.

### 4.1. Electrochemical Energy Storage (Nanoenergy) Application

When we look deep into the global energy outlook, we would see that the energy crises of the 21st century are a subject that has been discussed extensively by the scientific community. The problem aroused as a result of rapid depletion of fossil fuel reservoir. It is a common knowledge that fossil fuels as sources of energy are not environmental friendly, sustainable and generally non renewable [164]. Electrochemical devices such as supacapacitors, fuel cell, etc. were notably known as energy storage technologies used for electric power applications and the process is achieved through electrolysis. The chemical reaction that lead to electrical energy generation using fuel cells is considered safe and environmentally friendly as it does not produce any harmful gases (normally greenhouse gases) which are generally considered as detrimental to the atmosphere [5,15,165,166].

In spite of all these advantages, it is perturbing that fuel cells can only be effectively utilized when used with electrocatalyst. The electrocatalyst generally functioned to speed up oxygen reduction reactions in the cell [167]. Platinum is the most widely used catalyst for this purpose based on its excellent electrocatalytic activity when compared to other catalysts. However, the catalyst is expensive and non reliable. For this reason, researchers felt it was necessary to devise a novel ways to enhance their effectiveness and reduce production cost. Consequently, graphene and its derivatives, which are front liner carbon-based materials, are seen as an alternative to replace platinum.

The best advantage of carbon-based materials is that they are cheap as compared to metal catalyst like platinum. It has been reported that graphene quantum dots fabricated and self-assembled on graphene by hydrothermal process produced a new hybrid nanoplatelets with excellent catalytic properties even higher than that of commercial Pt/C in alkaline media [168]. Thus, the glaring prospect of fabricating carbon-based nanomaterials from renewable sources could possibly satisfy the energy demand of the 21st century, hence replacing the fossil fuel sources. The quality and the recharge time of supercapacitors, supercapacitors-battery hybrids and lithium batteries have been experimentally tested to be enhanced using carbon nanotubes [169,170,171], graphene and activated carbon [79], etc. It has been reported that porous graphene oxide when tested gave ultra-high capacitances of 283 Farads/gram and 234 Farads/cm^3^. Interestingly, by combining graphene with manganese dioxide (MnO_2_) capacitance of 1100 Farads/ cm^3^ was achieved. It is fascinating to note that these materials could possibly be used to store large amounts of electricity more efficiently produced from solar energy by more efficient solar panels. Similarly, the range, recharge time and reliability of electric vehicles will be enhanced and become much cheaper [172].

### 4.2. Biomedical Application

Due to its ultrahigh surface area (2630 m^2^/g) [159] and sp^2^ hybridized carbon area, grapheme (a very resourceful carbon nanomaterial) has been used as an excellent drug carrier to load large amount of drug molecules on both sides of the single atom layer sheet. The pioneer application for drug delivery was conceptualized by Dai et al. [173]. These authors reported that the physisorption via π-stacking can be applied for loading anticancer drugs SN38. It is noteworthy that graphene and other graphene-related and derived materials have now been extensively researched in the area of biomedicine and demonstrate very glaring future in this field.

In the area of biosensing technology, various biosensors based on different sensing mechanisms including optical and electrochemical signaling has been constructed from carbon nanomaterials (e.g., graphene based materials) [174]. Electrochemical technique has been reported as one of the most favorable method for biomolecule detection [175]. This owed to its operational simplicity, excellent sensitivity, inexpensiveness and quick response. Due to the alluring electrochemical properties of graphene, it is now used as electrode material to improve the detection of biomolecules. For instance, graphene has shown an excellent electrocatalytic activity toward H_2_O_2_, thus paving the way for this two-dimensional (2D) carbon crystal to be used as an electrode material for oxidase-based biosensors. It is clear that detection of glucose level in the patient’s body is clinically important for diagnosis of diabetes. Hence, electrochemical detection of glucose in the blood could be achieved using glucose oxidase as the mediator or detection element [176]. In one interesting research, Dan Du et al. developed a sensitive immunosensor for cancer biomarker based on dual signal amplification strategy of graphene sheets and multienzyme functionalized carbon nanospheres. Based on this finding, 7-fold increase in detection signal was achieved using the immunosensor as contrasted to the immunosensor without graphene modification and carbon nanospheres labeling [177]. Hence, this amplification approach could be essentially useful for clinical assessment of tumor biomarkers and point-of-care diagnostics.

## 5. Conclusions

In conclusion, this review has shown how carbon nanomaterials attracted attention of scientists from different walks of life which have drawn remarkable attention from fields ranging from chemistry, materials science and engineering to condensed-matter physics and from both industry and academia. Thus, the development of new carbon nanomaterials such as graphene, carbon nanotube etc. has undoubtedly promoted the science of nanotechnology in recent years; their unique properties were inestimable in a variety of technologies like electronics, optics, energy storage and many other applications. In general, all these have been achieved due to the uniqueness of these materials which is strongly related to their exceptional properties facilitated by their nanoscale structure which is unrivalled by any other known material.

Furthermore, from the structural illustration of some 0-, 1-, 2- and 3-dimensional carbon nanomaterials with sp^2^ and sp^3^ hybridization allotropes occurring in different crystallographic forms, it was inferred that carbon nanomaterials/allotropes encompasses or represent a set of materials mostly with different structure, morphology, and properties, but containing one major common element, carbon as the main building block of their structures. In general, the chemical versatility of carbon (especially its ability to catenate) serves as the driver that permits its conversion from sp^2^ to sp^3^ hybridization and remarkably binding with other atoms. This corroborates with the notion that carbon is one of the most amazing element in the Periodic Table as well as indispensable in our world.

It is noteworthy also that a remarkable progress regarding synthesis of carbon nanostructures from biomass resources has been reported. However, exploring the maximum potentials towards more greener and environment friendly synthesis methods and industrial scale production of these materials is undoubtedly necessary and can therefore be seen as the focal point of many researchers in science and technology in the 21st century. Consequently, this will help in reducing the much feared energy and environmental issues, more especially in the developing countries.

## 6. Outlook/Way Forward

In recent years, the special attention given to carbon nanomaterials, particularly graphene and its derivative products could be justified by the multitude of publications describing their distinctive properties which could be tailored toward a specific type of applications. Other determinants of the uniqueness of the carbon nanomaterials were the distinguished Nobel prizes awarded for the discovery of fullerene and graphene and the Kavli Prize awarded for outstanding contributions in advancing the knowledge and understanding of nanoscience and the discovery of carbon nanotubes. These have undoubtedly revealed the uniqueness of these carbon-based materials which is unmatched to any other conventional non carbon materials. However, despite all the glaring prospects shown by these carbon nanomaterials, a few drawbacks and limitations were also conceived in the industrial-scale production of some of these materials. For example, graphene oxide, a foremost carbon nanomaterials, which traced its history since 1898, and generally known as the most commonly resource and processable graphene precursor, however, its industrial commercialization has still remained a challenge as compared to CVD-graphene due to the influence of carbon or graphite source in the process of scale-up. For this reason, graphene oxide is predominantly processed together with other materials than as a complete product for a market with an open demand. In addition, its application is limited to where defect-free graphene is not important. In summary, like all other types of materials, carbon nanomaterials have their own few drawbacks. Interestingly, the positives far exceeded the negative ones as thoroughly expressed in this review. Nevertheless, their exact potentials and role could be further harnessed and clearly understood when those few drawbacks are addressed in the future studies. Categorically, a clear examination is essential of the limitations to which optimization of physicochemical and spectroscopic parameters of carbon nanostructures can be taken. More advanced application of these materials in areas like nanomedicine for improved HIV drug therapies; DNA-based single-electron electronic devices, brain-inspired devices for artificial systems, Super-powered bionic plants, light-seeking synthetic nano-robot, self-healable batteries, etc. should be extensively explored by the global scientific communities in this 21st century.

## Figures and Tables

**Table 1:** A literature report on preparation of graphene from various waste materials using different synthetic pathways.

Source/Precursors	Materials	Reaction Condition: Catalyst and Additives	Reactors	Product/Results	Reference
Biomass wastes	Leaf, chicken bone, baggase, wood, industrial soot, newspaper	Chemically derived method; H_2_SO_4_	Not specified	rGO sheets	[82]
Biomass wastes	Crustacean skin wastes	Catalyst free	Unspecified	Monolayer N-doped-graphene: large size, 99% transmittance	[125]
Biomass wastes	Coconut shell	FeCl_3_ and ZnCl_2_	Chemical vapor deposition., the tube was not specified	PGNs: highly interconnected porous structure, good energy density, large surface area, capacitance	[126]
Biomass wastes	Grass blades, dog feces, cockroach legs, waste cookies and chocolate	Cu foil	Quartz tube	Monolayer graphene: high quality, low defects, 97% transmittance	[84]
Biomass wastes	Dead neam leaves	Pyrolysis in a tube furnace, post-treated with chemical solutions		GQDs: incredible florescence, biocompatibility, size effect on band gap	[127]
Waste plastics	PTFE (SiC)	Catalysts free. The synthetic pathway used to produce graphene does not require an external energy source. It took place in a self-sustained synergistic way.	High-pressure stainless steel reactor	Graphene sheets coated on porous carbon particles with large accessible surface area; with a 28% carbon yield	[128]
Waste plastics	PPMA; sapphire (11–20) substrates as a carbon source	Pyrolysis; Cu thin layer	CVD, tube not specified	Thin films of graphene	[129]
Solid waste plastics	PE (86%)–PS (14%)	Cu foil; Ambient pressure (AP) CVD process	AP-CVD system with a quartz tube	Lower rate of pyrolysis and injection; higher rate of injection: Large hexagonal shaped single graphene crystal; bilayer or multilayer graphene, respectively.	[130]

**Table 2:** A literature description on preparation of activated carbon from various biomass residues by carbonization and different activation conditions.

S/N	Precursors/Raw Materials	Carbonization Atmosphere	Activation Conditions	Chemical Agents	Supplementary Explanation	References
1	Almond tree pruning and Almond shell	N_2_, 600 °C/1 h	850 °C/30 min	Steam	The diluted steam was physically in touch with the biochars accordingly	[134]
2	Bagasse	N_2_, 500 °C/1 h	N/A	ZnCl_2_	Single step carbonization-activation, impregnation	[135]
3	Bamboo	N_2_, 400–500 °C/2 h	800 °C/2 h	HCl	Impregnated with 0.1 M HCl	[136]
4	Coconut shell	N_2_, 250–750 °C/1 h	500–900 °C/15 min	K_2_CO_3_	Chemically mediated activation, impregnation ratio 1:1	[137]
5	Coconut shell	N_2_, 400–800 °C/1 h	800 °C/60–270 min	Steam	Chars get in touch with N_2_ and H_2_O afterward	[138]
6	Coconut shell	N_2_, 850 °C/1 h	850 °C/5–80 min	CO_2_	One step Pyrolysis/activation	[139]
7	Coffee waste	N_2_, 700 °C	700 °C/2–3 h	CO_2_/ZnCl_2_ and KOH	Heating rate of 10 °C/min; Impregnation ratio 2:1 to 3:1	[140]
8	Date tree frond	N_2_, 400 °C/3 h	N/A	H_3_PO_4_	Single step carbonization-activation	[141]
9	Ground nut shell	N_2_, 800 °C/5 min	N/A	ZnCl_2_	One step and two step activation, respectively	[142]
10	Ground nut shell	N_2_, 800 °C/5 min	N/A	H_3_PO_4_	One step and two step activation, respectively	[142]
11	Ground nut shell	N_2_, 800 °C/5 min	N/A	KOH	Both one step and two step activation	[142]
12	Hazelnut Baggase	N_2_, 500–700 °C/2h	N/A	ZnCl_2_	One step carbonization/activation	[143]
13	Hazelnut Baggase	N_2_, 500–700 °C/2 h	N/A	KOH	One step carbonization/activation	[143]
14	Kenaf Fibre	N_2_, 400 °C/2 h	700 °C/1 h	CO_2_/KOH	Impregnation of the char was done via KOH at 1:4 ratio	[144]
15	Mango seed shell	N_2_, 500 °C/1 h	N/A	ZnCl_2_	One step carbonization-activation, impregnation	[145]
16	Neem Husk	N_2_, 200–500 °C/10 min	N/A	KOH	One step carbonization-activation, most favorable at 350	[146]
17	Olive waste cake	N_2_, 350–650 °C/2 h	N/A	H_3_PO_4_	single step carbonization-activation	[147]
18	Oil palm shell	N_2_, 500 °C/3 h; CO_2_/1 h	500 °C/1 h	ZnCl_2_/CO_2_	Chemical activation coupled by physical activation; N_2_, gas was later replaced by flowing CO_2_ gas for one hour.	[148]
19	Palm kernel shell	N_2_, 400 °C/1 h	800–1000 °C; 15–40 min	KOH	Carbonization followed by impregnation for 2 h	[149]
20	Palm shell	N_2_, 400–800 °C/3 h	400–800 °C/90 min	CO_2_/ZnCl_2_	Physical activation, 65% ZnCl_2_	[150]
21	Palm oil trunk	N_2_, 500 °C/3 h; CO_2_/1 h	500 °C/1 h	H_3_PO_4_/CO_2_	The ratio of the acid to the precursor of 0.9 was used, followed by carbonization and activation using CO_2_	[97]
22	Rice husk	N_2_, 500 °C/1 h	N/A	ZnCl_2_	One step carbonization-activation, impregnation	[151]
23	Walnut shell	N_2_, 600 °C/1 h	850 °C/30 min	Steam	Chars were subsequently in contact with diluted steam	[152]

**Table 3:** Comparison of some properties of various carbon nanomaterials [160].

Carbon Nanomaterials	Dimensions	Hybridization	Experimental Specific Surface Area (m^2^ g^−1^)	Thermal Conductivity (W m^−1^ K^−1^)	Electrical Conductivity (S cm^−1^)	Tenacity	Hardness
Graphite	3	sp^2^	~10–20	Anisotropic: 1500–2000, 5–10	Anisotropic: 2–3 × 10^4^	Flexible, non-elastic	High
Graphene	2	sp^2^	~1500	4840–5300	~2000	Flexible, elastic	Uppermost (for single layer)
Carbon nanotube	1	mostly sp^2^	~1300	3500	Structure-dependent	Flexible, elastic	High
Fullerene	0	mostly sp^2^	80–90	0.4	10^−10^	Elastic	High

**Table 4:** Comparison of some properties of the two renowned allotropes of carbon; graphite and diamond.

Properties	Graphite	Diamond
Crystal system and form	Hexagonal; substantial lamellar veins and earthy masses	Isometric; cubes and octahedrons
Specific Gravity	2.2	3.5
Density (g/cm^3^)	2.25	3.52
Color/Appearance	Grey black, Black silver, opaque shiny	Variable-pale yellows, browns, grays, and also white, blue, black, reddish, greenish, colorless and sparkling
Hardness (Mohs)/Field indicator	1–2; Soft, slippery, soapy, greasy luster, density and streak	10; Very Hard (a hardest substance known)
Luster	Metallic to dull	Adamantine to waxy
Cleavage	Perfect in 1 direction	Perfect in 4 directions forming octahedrons
Transparency	Crystals are opaque	Crystals are transparent to translucent in rough crystals
Fracture	Flaky	Conchoidal
Electrical and Heat conductivity (E&H)	Good conductor of both E&H	Poor electrical conductor; good thermal conductor
Burning in the air	At about 700 °C	Most readily at about 900 °C
